# The exploration of disease-specific gene regulatory networks in esophageal carcinoma and stomach adenocarcinoma

**DOI:** 10.1186/s12859-019-3230-6

**Published:** 2019-12-30

**Authors:** Guimin Qin, Luqiong Yang, Yuying Ma, Jiayan Liu, Qiuyan Huo

**Affiliations:** School of Computer Science and Technology, Xidian University, Xi’an, 710071 China

**Keywords:** Esophageal carcinoma, Stomach adenocarcinoma, Molecular mechanism, Feed-forward loop, Random walk with restart

## Abstract

**Background:**

Feed-forward loops (FFLs), consisting of miRNAs, transcription factors (TFs) and their common target genes, have been validated to be important for the initialization and development of complex diseases, including cancer. Esophageal Carcinoma (ESCA) and Stomach Adenocarcinoma (STAD) are two types of malignant tumors in the digestive tract. Understanding common and distinct molecular mechanisms of ESCA and STAD is extremely crucial.

**Results:**

In this paper, we presented a computational framework to explore common and distinct FFLs, and molecular biomarkers for ESCA and STAD. We identified FFLs by combining regulation pairs and RNA-seq data. Then we constructed disease-specific co-expression networks based on the FFLs identified. We also used random walk with restart (RWR) on disease-specific co-expression networks to prioritize candidate molecules. We identified 148 and 242 FFLs for these two types of cancer, respectively. And we found that one TF, *E2F3* was related to ESCA, two genes, *DTNA* and *KCNMA1* were related to STAD, while one TF *ESR1* and one gene *KIT* were associated with both of the two types of cancer.

**Conclusions:**

This proposed computational framework predicted disease-related biomolecules effectively and discovered the correlation between two types of cancers, which helped develop the diagnostic and therapeutic strategies of Esophageal Carcinoma and Stomach Adenocarcinoma.

## Background

Esophageal Carcinoma (ESCA) and Stomach Adenocarcinoma (STAD) are two types of cancer in the digestive tract. ESCA ranks sixth in its cancer-related mortality rate [1, 2]. ESCA is classified histologically as esophageal adenocarcinoma (EAC) and esophageal squamous cell carcinoma (ESCC) [3]. Stomach Adenocarcinoma is one of common malignancies of digestive tract [4, 5]. Despite the advances in the treatment of STAD, the 5-year survival rate is 5~15% [6]. Both ESCA and STAD belong to digestive tract cancer, and the sites of their incidence are very close, so it is significant to explore the molecular mechanisms and the relationship between these two types of cancer.

Recently, comprehensive analysis of molecular characteristics of many types of cancer was performed, including STAD and ESCA. For example, Yin et al. conducted a case-control study based on their own patients and provided the first evidence that RANK rs1805034 T>C polymorphism was associated with susceptibility of ESCA [2]. A study by Pan et al. showed that lncRNA *CASC9* in ESCA tissue was up-regulated [7]. *SLC52A3* was proved to be useful for proliferation and colony formation of ESCA [8]. Baffa R et al. focused on loss of heterozygosity for chromosome 11 in STAD as early as 1996 [9]. An allelotype analysis was performed to identify chromosomal regions which were frequently deleted in STAD [10]. Korean researchers analyzed protein expression profiles of five STAD suppressor genes [11]. The Cancer Genome Atlas (TCGA) Research team performed a comprehensive molecular analysis of 559 patients of Stomach Adenocarcinoma and Esophageal Carcinoma, and found that EAC was closely resembled Stomach Adenocarcinoma by analyzing mRNA expression, DNA methylation and SCNA data [12]. In a recent study, the researchers questioned the use of *PD-L1* as a biomarker in both of ESCA and STAD [13]. Most of the studies focused on ESCA or STAD separately, ignoring their potential common molecular characteristics, so it is of great importance to compare these two types of cancer.

Gene expression is regulated by many factors, among which TFs and miRNAs are two most important factors, and a feed-forward loop (FFL) consisting of two regulation factors and a common target gene plays an essential role in many biological processes [14]. FFLs were proved to be relevant to diseases, so some studies were performed to identify significant FFLs in complex diseases, including schizophrenia, Glioblastoma, T-cell acute lymphoblastic leukemia and so on [15–17]. There were also some studies identifying common FFLs in pan-cancer [18, 19]. Besides, TF-miRNA-lncRNA FFLs were identified [20]. However, FFLs in ESCA and STAD have not been studied yet as far as we know.

In this paper, we investigated the common and distinct regulatory properties of ESCA and STAD. Firstly, we identified miRNA-TF-gene FFLs by integrating gene/miRNA expression profiles and transcriptional/post-transcriptional regulation pairs. Then, we built and analyzed disease-specific co-expression networks based on the identified FFLs. Finally, we prioritized candidate disease-related biomolecules based on their scores.

## Results

### Overview of the proposed computational framework

**Fig. 1:**
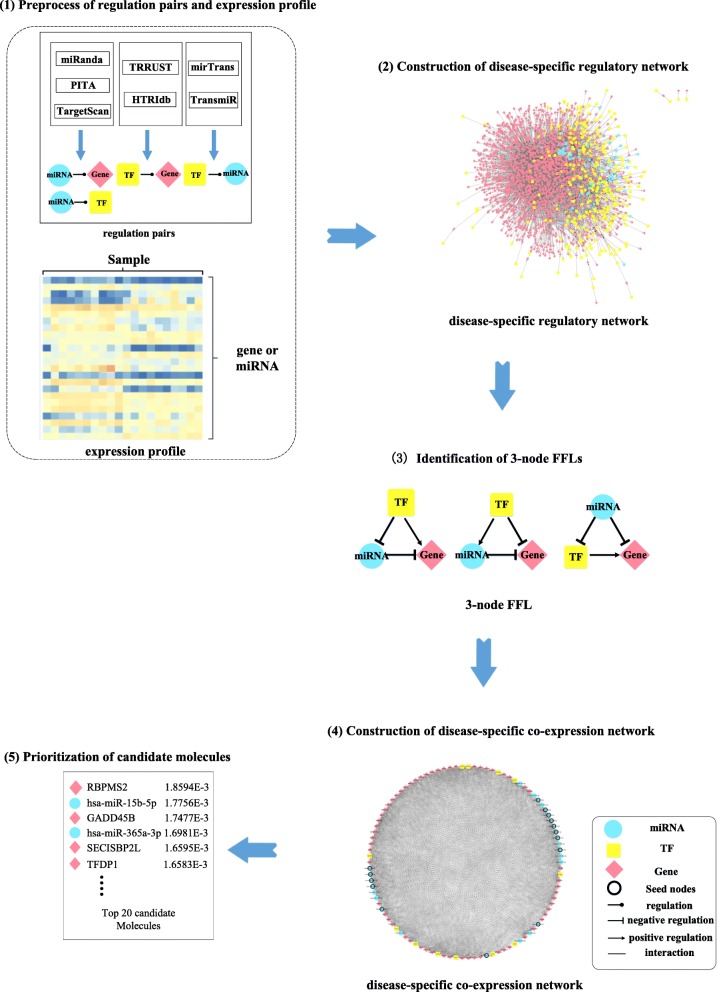
Flowchart of the proposed computational framework. (*1*) Preprocessing of regulation pairs and expression profiles (*2*) Construction of disease-specific regulatory networks (*3*) Identification of 3-node FFLs (*4*) Construction of disease-specific co-expression networks (*5*) Prioritization of candidate molecules

The proposed computational framework consisted of the following five steps (Fig. 1), and we described each step briefly.

- Step 1. Preprocessing of regulation pairs and expression profiles. We combined TF-target pairs and miRNA-target pairs from different algorithms and databases, and dealt with noisy data. For expression profiles, we filtered out the genes with low expression level and miRNAs with many missing values. We also calculated differentially expressed genes and miRNAs for ESCA and STAD using Limma [21] with adjusted *p*-value smaller than 0.05 and |log_2_FC| greater than 1.
- Step 2. Construction of disease-specific regulatory networks. We constricted the target genes as differentially expressed genes and the miRNAs as differentially expressed miRNAs. And then we contained the regulation pairs whose spearman correlation coefficient (SCC) was greater than 0.3. As a result, the disease-specific regulatory networks for ESCA and STAD were constructed.
- Step 3. Identification of 3-node FFLs. Three types of typical FFLs were identified from the disease-specific regulatory networks for ESCA and STAD.
- Step 4. Construction of disease-specific co-expression networks. We calculated SCC for each pair of the molecules in the identified FFLs and then constructed diseased-specific co-expression networks using those pairs with coefficient absolutely greater than a predefined threshold.
- Step 5. Prioritization of candidate molecules. Random walk with restart (RWR) was used to calculate the score of each biomolecule in the co-expression networks. The higher the score was, the more likely the biomolecule was a disease-related molecular.

### Disease-specific regulatory network analysis

First of all, we combined differentially expressed molecules with preprocessed regulation pairs. And then we calculated SCC for each regulation pair. We chose the threshold with correlation as 0.3 and *p*-value as 0.05 so that we obtained the disease-specific regulatory networks for ESCA and STAD. The results were shown in Table 1.

**Table 1:** Disease-specific regulatory networks for ESCA and STAD

Cancer	Relationship	RegulationType	Pairs	miRNAs	TFs	Genes
ESCA	TF-gene	positive	2096	–	154	1353
		negative	1383	–	101	1032
	TF-miRNA	positive	136	54	51	–
		negative	68	31	36	–
	miRNA-gene	negative	1674	48	–	579
	miRNA-TF	negative	444	47	165	–
STAD	TF-gene	positive	2454	–	199	1534
		negative	1307	–	153	942
	TF-miRNA	positive	154	70	51	–
		negative	165	78	45	–
	miRNA-gene	positive	3689	80	–	847
	miRNA-TF	negative	1304	80	277	–

**Fig. 2:**
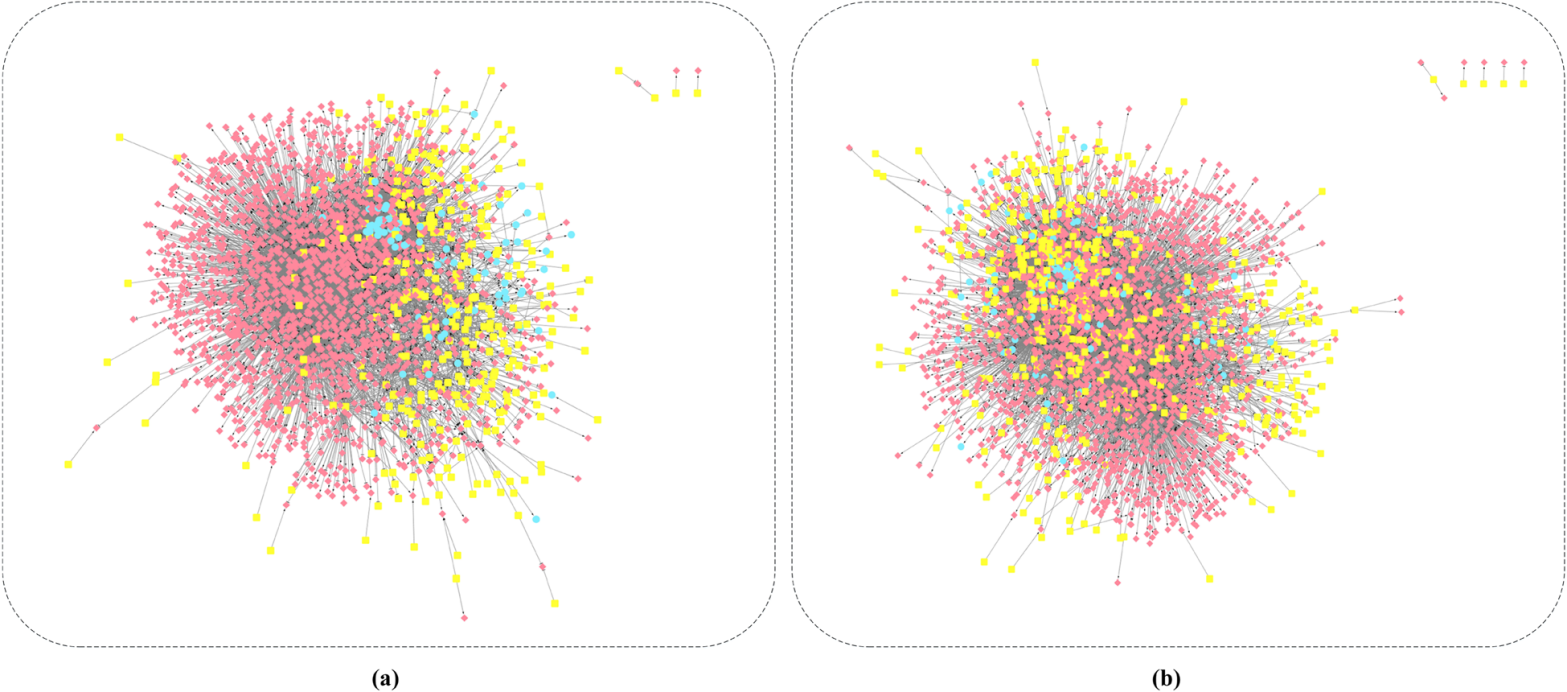
Disease-specific regulatory networks. **a** ESCA **b** STAD

There were 79 miRNAs, 325 TFs, 1830 genes, and 5801 regulation pairs in ESCA-specific regulatory network (Fig. 2a). And the obtained STAD-specific regulatory network consisted of 116 miRNAs, 461 TFs, 2093 genes, and 9037 regulation pairs (Fig. 2b).

### MiRNA-TF-gene FFLs

We identified three categories of FFLs from the disease-specific regulatory networks for ESCA and STAD, respectively. And we named the FFLs identified from the ESCA-specific regulatory network as ESCA-specific FFL and the FFLs identified from the STAD-specific regulatory network as STAD-specific FFL. The results were summarized in Table 2.

**Table 2:** The number of FFLs identified

Cancer	FFL	FFLs	miRNAs	TFs	Genes
ESCA	TFP-FFL	7	3	2	7
	TFN-FFL	14	6	4	13
	miRNAN-FFL	127	19	8	46
STAD	TFP-FFL	38	12	9	21
	TFN-FFL	46	16	9	32
	miRNAN-FFL	158	38	21	48

**Fig. 3:**
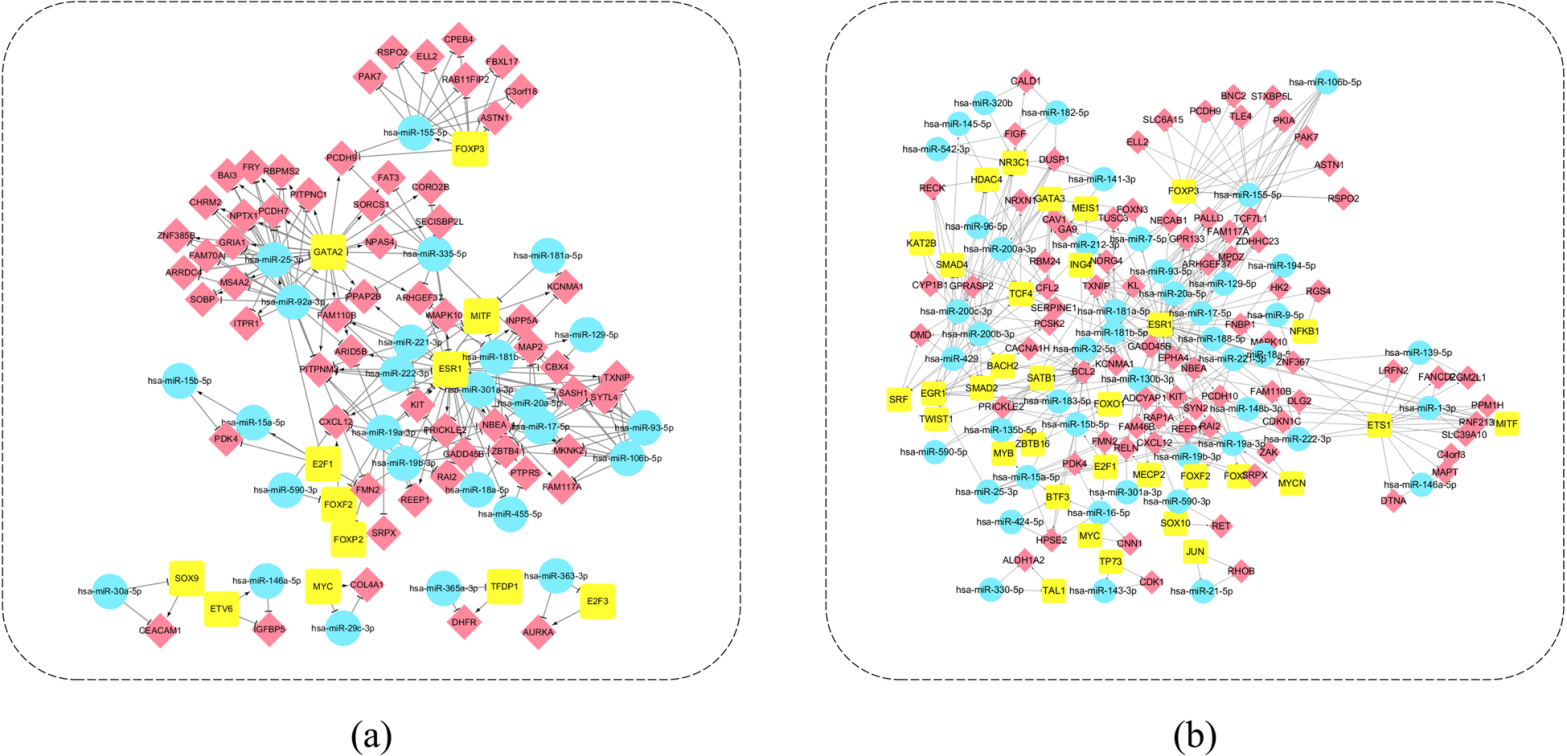
Disease-specific FFL networks. **a** ESCA **b** STAD

There were 7 TFP-FFLs, 14 TFN-FFLs and 127 miRNAN-FFLs for ESCA, respectively. An ESCA-specific regulatory network was constructed based on the identified FFLs, which consisted of 26 miRNAs, 12 TFs, 60 genes and 240 regulation pairs (Fig. 3a).

What’s more, there were 38 TFP-FFLs, 46 TFN-FFLs and 158 miRNAN-FFLs for STAD. A STAD-specific FFL network was constructed based on the identified FFLs, which consisted of 47 miRNAs, 31 TFs, 87 genes and 401 regulation pairs (Fig. 3b).

For both of ESCA and STAD, the number of miRNAN-FFL was the largest one, which meant that this FFL model was the most common regulatory pattern, and the genes were mainly down-regulated.

**Fig. 4:**
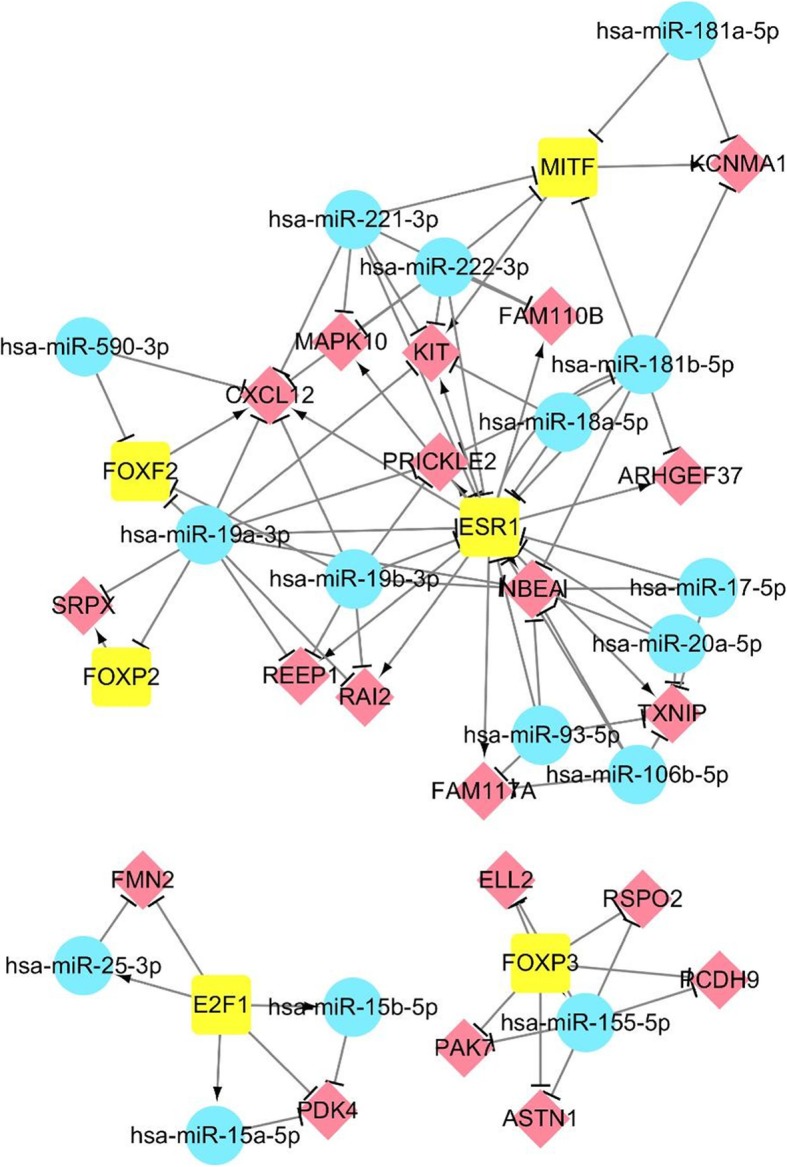
The common regulatory network for ESCA and STAD

We further investigated the common FFLs in the ESCA-specific FFL and STAD-specific FFL, and found that there were 3 TFP-FFLs, 8 TFN-FFLs, and 41 miRNAN-FFLs. It is exciting that STAD and ESCA shared so many FFLs, which provided a strong evidence for the potential closely relationship between STAD and ESCA. We further constructed a regulatory network for ESCA and STAD based on these common FFLs (Fig. 4), which was made up of three subnetworks. What’s more, there were 16 miRNAs, 6 TFs, 20 genes and 90 regulation pairs in this common network.

**Fig. 5:**
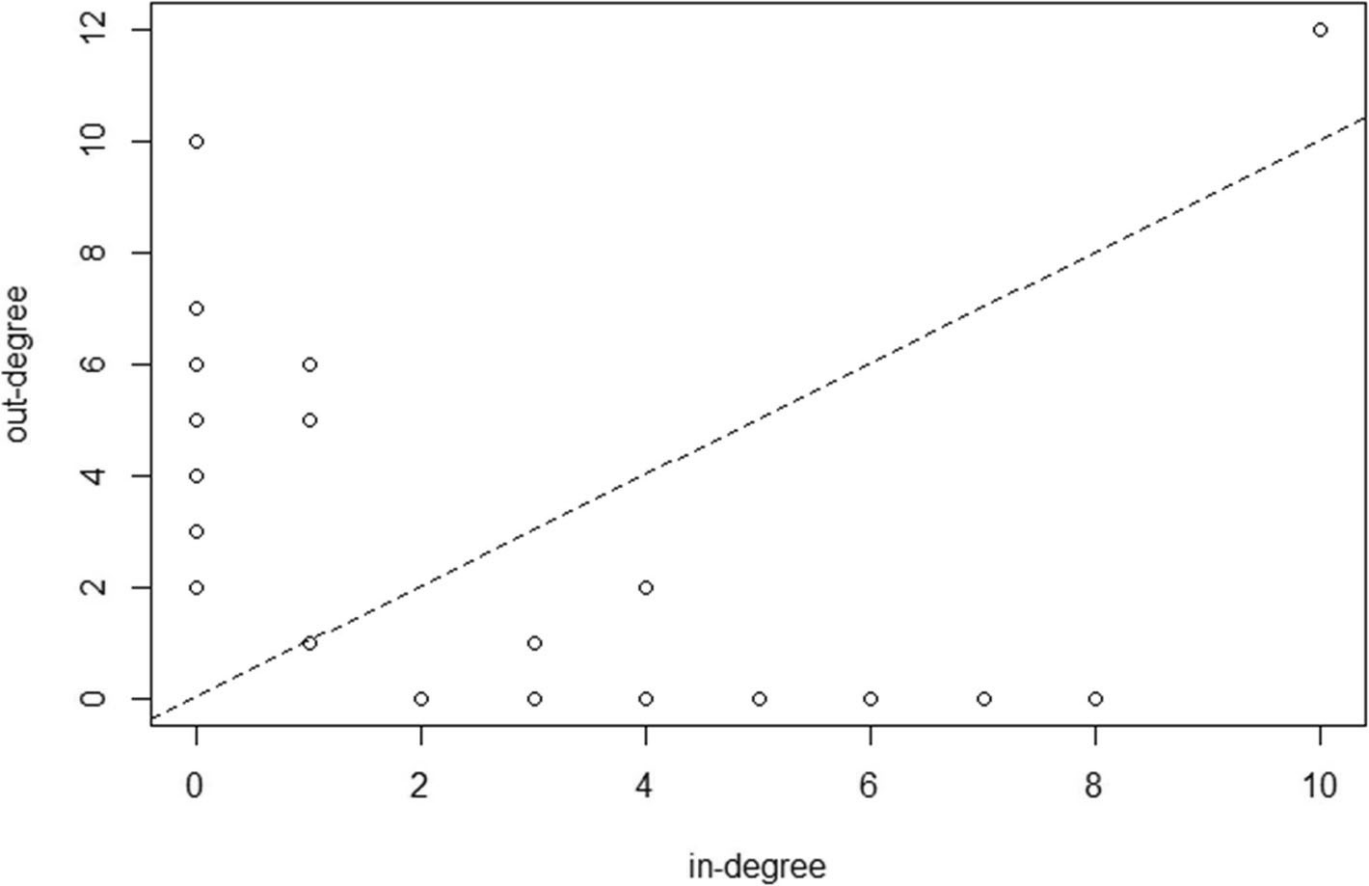
The in-degree and out-degree distribution of the regulatory network for ESCA and STAD

We investigated the in-degree and out-degree properties of the regulatory network. Figure 5 showed the in-degree and out-degree distribution of this network. We found that the nodes which only had in-degree were all genes, and the number of nodes was 20. And there were 11 miRNAs and 2 TFs which only had out-degree in this network. 5 miRNAs and 4 TFs not only had in-degree but also had out-degree. Among these nodes, one TF *ESR1* had highest in-degree and highest out-degree. We found it was related to both of ESCA and STAD. Genetic variations in *ESR1* were associated with an increased risk of ESCA [22]. *ESR1* regulated stomach-specific tumor suppressor gene *TFF1*, further influenced the development of STAD [23].

### Gene set enrichment analysis

Gene set enrichment analysis is a meaningful way to understand the functions of genes in living cells. We applied the online tool DAVID [24] to perform gene set enrichment analysis for those genes. With the DAVID online tool, we set the threshold *p*-value as 0.05 and then obtained a list of entries.

For the STAD-specific FFL, there were 114 biomolecules, including 33 TFs and 81 genes, and they were enriched in a total of 191 annotation entries, including 162 GO terms and 29 BIOCARTA and KEGG pathways. For the ESCA-specific FFL, there were 72 biomolecules, including 12 TFs and 60 genes, and analyzed them with DAVID online tool. They were resulting in a total of 53 annotation entries, including 42 GO terms and 11 BIOCARTA and KEGG pathways. An additional file showed this in more detail [see Additional file 1].

We further found 32 common entries, including 27 GO terms and 5 KEGG pathways. We selected 10 enrichment entries for further analysis (Table 3). Among these entries, the disease-specific FFLs had similar number of genes. For biological processes, the genes in both types of disease-related FFLs were enriched in negative regulation of transcription from RNA polymerase II promoter, which also indicated that these genes played an important role in transcriptional regulation. For molecular components, the genes were enriched in the nucleoplasm. The nucleus was a necessary component in the cell, which also showed that these genes were vital and had an indispensable effect on the cell body and even the living body. For molecular function, the genes were enriched in transcription factor activity, sequence-specific DNA binding.

**Table 3:** The common enrichment entries for ESCA and STAD

Category	Term	Genes in ESCA	Genes in STAD
BP (GO:0000122)	Negative regulation of transcription from RNA polymerase II promoter	12	27
BP (GO:0045944)	Positive regulation of transcription from RNA polymerase II promoter	11	28
BP (GO:0009791)	Post-embryonic development	3	5
CC (GO:0005654)	Nucleoplasm	18	28
CC (GO:0005667)	Transcription factor complex	5	11
CC (GO:0043234)	Protein complex	6	10
MF (GO:0005515)	Protein binding	42	72
MF (GO:0003700)	Transcription factor activity, sequence-specific DNA binding	12	26
MF (GO:0019901)	Protein kinase binding	6	7
KEGG (hsa04110)	Cell cycle	5	7

For biological pathways, genes were enriched in the cell cycle which was a continuous process passing from one generation to the next. The enriched members of this pathway for the ESCA-specific FFL were 4 TFs, *E2F1*, *E2F3*, *MYC*, *TFDP1* and 1 gene *GADD45B*. And the enriched members of this pathway for the STAD-specific FFL were 4 TFs, *E2F1*, *SMAD4*, *SMAD2*, *MYC* and 3 genes which included *CDKN1C, CDK1*, *GADD45B*. The common members which were 2 TFs, *E2F1*, *MYC* and 1 gene *GADD45B,* were all related to both of two types of cancer. All these categories showed that these enriched genes may have a critical impact on the emergence and development of the disease.

### Co-expression network analysis

**Fig. 6:**
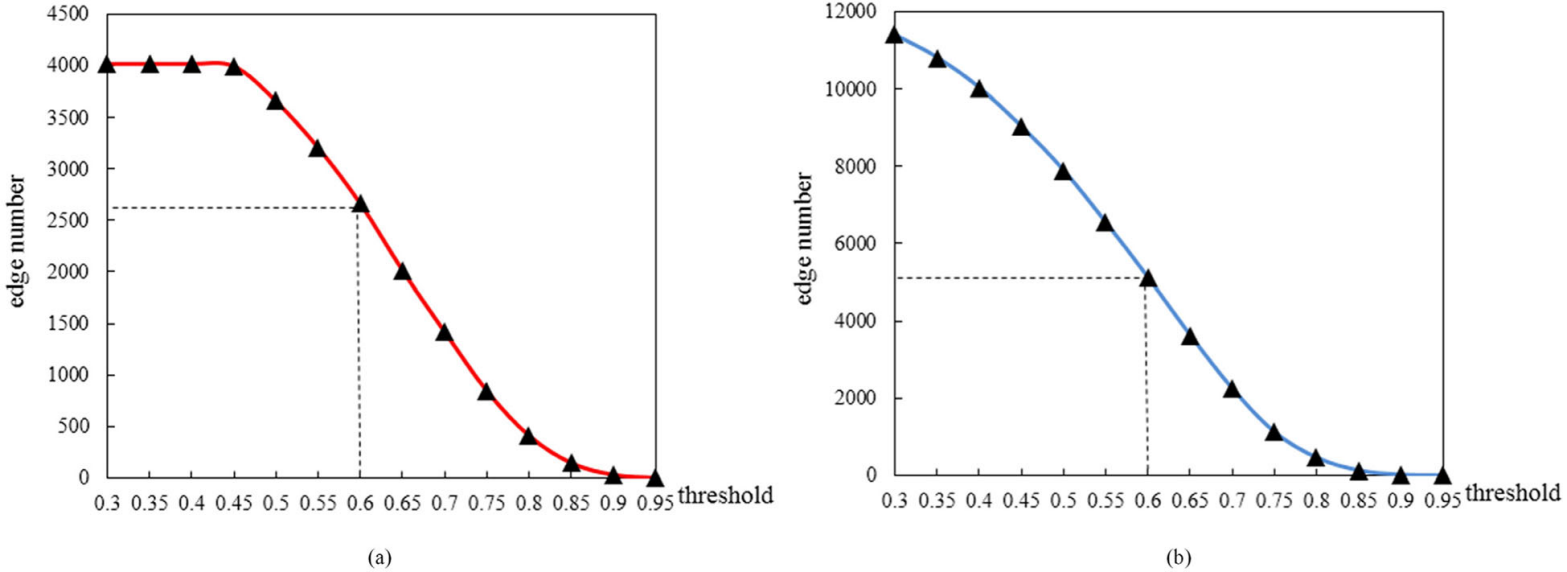
The relationship between thresholds and edge numbers. **a** ESCA **b** STAD

We further focused on the molecules in the identified FFLs and investigated their SCC for all pairs of molecules to build disease-specific co-expression networks. We observed different sizes of co-expression network for different thresholds. Figure 6 showed the relationship between thresholds and the size of co-expression networks for STAD and ESCA. This relationship could be fitted to a cubic function. The cubic function’s inflection point is very meaningful. Before this point, the network size decreases sharply with the increase of threshold, and after this point, the network size decreases slowly with the increase of threshold. So the network at this point is more representative. The thresholds corresponding to the inflection points of the fitting functions of disease-specific co-expression networks for ESCA and STAD are about 0.6. Consequently, we chose 0.6 as the cut-off value in these two networks, and meanwhile *p*-value was less than 0.05. Finally, there were 98 nodes with 2666 pairs and 158 nodes with 5117 pairs in the disease-specific co-expression networks for ESCA and STAD, respectively.

Specifically, compared with the STAD-specific FFL, there were 3 molecules lost in the STAD-specific co-expression network. Because these 3 molecules had weak association with the other molecules.

### Random walk with restart in co-expression network analysis

We investigated the molecules in the disease-specific co-expression networks for ESCA and STAD. We collected 15 and 39 disease-related molecules in disease-specific co-expression networks for ESCA and STAD, respectively, as we have mentioned in Methods. Taking these disease-related molecules as seed nodes, and the other 83 and 119 molecules as candidates, we ran RWR on the disease-specific co-expression networks for STAD and ESCA, respectively. As a result, we could obtained the scores for each candidate molecule. The higher the score was, the more relevant the candidate molecules were with the specific disease.

In order to evaluate and select the appropriate restart probability *r*, the AUC value of sorting correctness was calculated when the value *r* varies from 0.1 to 0.9 step by 0.1 using leave-one-out cross validation, following the method proposed by RWRMDA [25]. Table 4 shows the relationship between the restart probability and the corresponding AUC. And we found that the AUCs for both of two types of cancer were really great when *r* varied from 0.1 to 0.9. When the restart probability is 0.9, we obtained the highest AUC, so we assigned 0.9 to the restart probability.

**Table 4:** The relationship between the restart probability and the corresponding AUC

* r*	0.1	0.2	0.3	0.4	0.5
*ESCA*	0.5550	0.6137	0.6442	0.6667	0.6796
*STAD*	0.6124	0.6660	0.7128	0.7468	0.7778
* r*	0.6	0.7	0.8	0.9	
ESCA	0.6996	0.7060	0.7181	0.7229	
STAD	0.7992	0.8214	0.8375	0.8500	

We listed the top 20 candidate molecules for both ESCA and STAD (Tables 5 and 6). Also there were 12 out of 20 candidate molecules supported by literature in PubMed for ESCA (Table 5), and 13 out of 20 candidate molecules were supported by literature in PubMed for STAD (Table 6). And details of the all candidate molecules can be showed in additional file [see Additional file 2]. These results showed that our analysis was reliable in a certain degree. And the molecules un-supported by literature may be potential disease-related molecules.

**Table 5:** Top 20 candidate molecules for ESCA

Ranking	Molecule	Score(10^−3^)	PMID
1	*RBPMS2*	1.8594	29301256
2	*hsa-miR-15b-5p*	1.7756	25943911
3	*GADD45B*	1.7477	16026601
4	*hsa-miR-365a-3p*	1.6981	–
5	*SECISBP2L*	1.6595	–
6	*TFDP1*	1.6582	14618416
7	*hsa-miR-222-3p*	1.6557	26258795
8	*AURKA*	1.6530	24953013
9	*SORCS1*	1.6250	–
10	*hsa-miR-106b-5p*	1.6121	27619676
11	*hsa-miR-18a-5p*	1.5999	23643275
12	*TXNIP*	1.5953	29934340
13	*ARHGEF37*	1.5939	–
14	*FBXL17*	1.5910	–
15	*E2F3*	1.5658	28751461
16	*hsa-miR-17-5p*	1.5597	28002789
17	*RAI2*	1.5583	–
18	*ITPR1*	1.5563	–
19	*SOX9*	1.5302	29936467
20	*MITF*	1.5277	–

**Table 6:** Top 20 candidate molecules for STAD

Ranking	Molecules	Score(10^−3^)	PMID
1	*CNN1*	1.5602	–
2	*hsa-miR-15a-5p*	1.5177	26894855
3	*REEP1*	1.2745	–
4	*DTNA*	1.2160	27858295
5	*FOXP2*	1.1572	27382302
6	*hsa-miR-188-5p*	1.1426	29471891
7	*hsa-miR-590-3p*	1.1182	29516678
8	*DLG2*	1.0800	–
9	*KCNMA1*	1.0594	28231797
10	*RELN*	1.0589	19956836
11	*CFL2*	1.0466	29342841
12	*hsa-miR-590-5p*	1.0191	27757042
13	*NECAB1*	1.0111	–
14	*PRICKLE2*	0.9895	16273260
15	*TUSC3*	0.9824	22447362
16	*hsa-miR-424-5p*	0.9587	27655675
17	*GPRASP2*	0.9453	–
18	*MEIS1*	0.9435	28545608
19	*MAPK10*	0.9298	–
20	*PCSK2*	0.9209	–

Furthermore, we investigated the molecules in the ranking lists, and found four interesting genes *RAI2*, *KCNMA1*, *NBEA* and *KIT*. These four genes ranked relatively closely in their disease-specific ranking list, and ranked in the first half among the whole candidate molecules (Table 7). And *KCNMA1*, *NBEA* and *KIT* were all related with STAD supported by published literatures [26–28], and *KIT* was also related with ESCA [29]. According to the enrichment analysis results of ESCA and STAD, *RAI2*, *KCNMA1* and *KIT* are involved in protein binding. *NEBA* is involved in protein kinase binding.

**Table 7:** The common molecules supported by PubMed

Candidate Molecule	ESCA_Ranking	STAD_Ranking	ESCA_PMID	STAD_PMID
*RAI2*	17	36	–	–
*KCNMA1*	36	9	–	28231797
*NBEA*	37	52	–	28035468
*KIT*	41	50	21626441	25741136

**Fig. 7:**
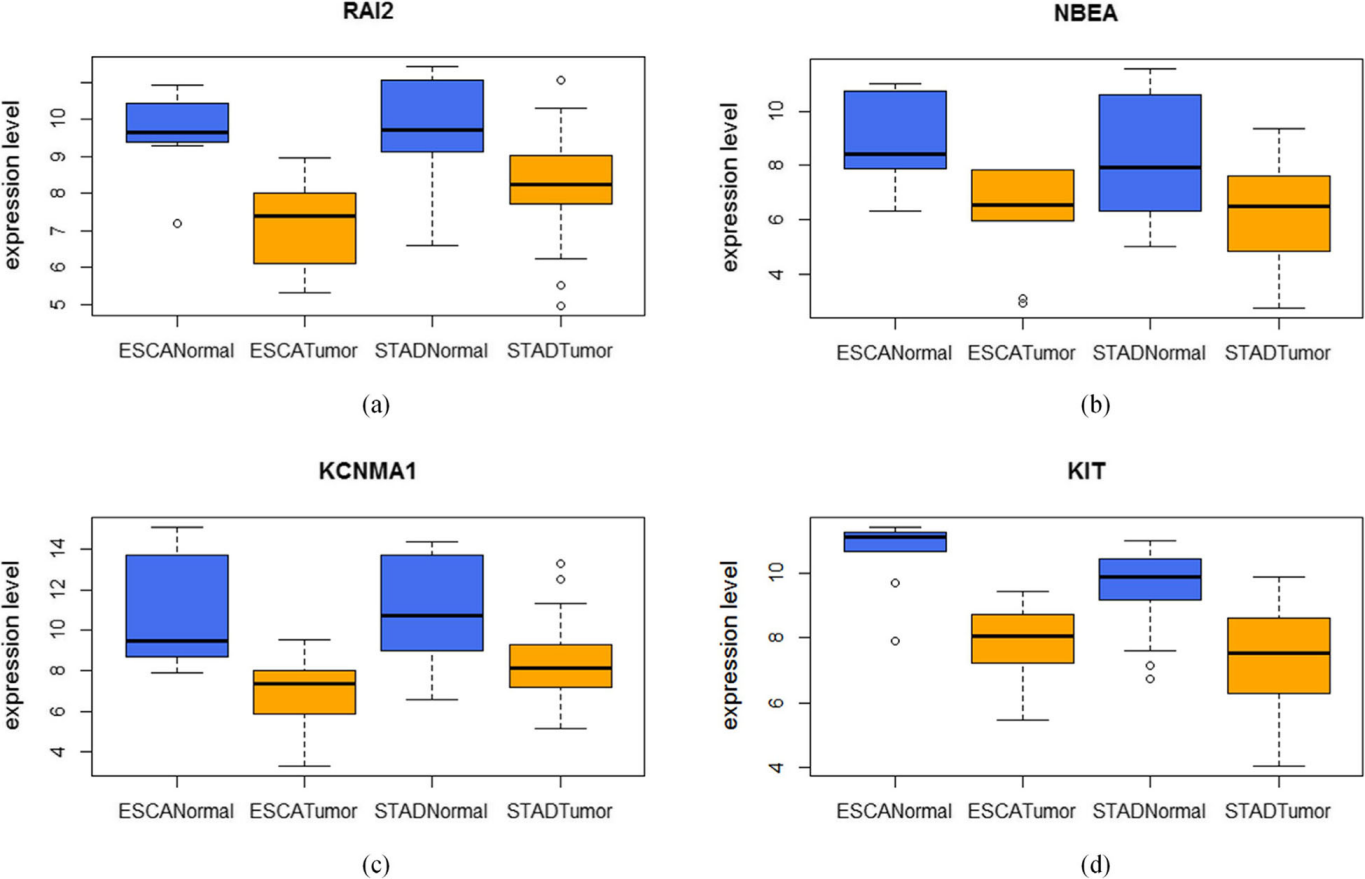
The expression levels of four genes in different samples. **a** *RAI2* **b** *NBEA* **c** *KCNMA1* **d** *KIT*

Then we investigated the expression level of these 4 genes. And all these genes were down-regulated in these two types of cancer, as shown in Fig. 7, which showed that these two types of cancer shared similar molecular characteristics.

## Discussion

We identified 148 and 242 FFLs for ESCA and STAD, respectively, and 52 FFLs were common for both of the two types of cancer, which meant that ESCA and STAD shared common regulatory properties. Gene set enrichment analysis for the genes in the FFLs also showed that they share many functional entries, including GO terms and biological pathways. For the top 20 candidate molecules in the ranking list, we validated 13 and 12 molecules in literature for ESCA and STAD, respectively, which also showed that our analysis is effective. We also investigated four genes, *RAI2, KCNMA1, NBEA,* and *KIT*, in the two ranking lists, and their potential functions for these two types of cancer. In all, we found that ESCA and STAD were close related with each other from the gene regulation prospect.

## Conclusions

We proposed a computational framework to investigate the regulatory properties of ESCA and STAD. In detail, we integrated gene/ miRNA expression profiles and TF/miRNA-target pairs from different data sources. Then we constructed disease-specific regulatory networks for ESCA and STAD, respectively, and identified FFLs from these two regulatory networks. We further analyzed the molecules in the identified FFLs and built two disease-specific co-expression networks. Finally, we prioritized candidate disease molecules using random walk with restart in these two disease co-expression networks. The results showed that ESCA and STAD shared common gene regulatory properties and molecular characteristics.

In this study, we performed a systematic analysis of gene regulatory properties of two types of cancer in the digestive tract. We focused on three points: firstly, we compared the molecular mechanisms of these two types of cancer, ESCA and STAD. Secondly, we built disease-specific regulatory networks and identified FFLs. Thirdly, we built disease-specific co-expression networks and predicted candidate molecules with RWR.

However, there are some problems in our study. Firstly, our analysis was heavy influenced by the incomplete and noise public data. Secondly, more omics data should be included to provide a more comprehensive model for the complex biological system.

## Methods

### Data source and pre-processing

#### Transcriptional/post-transcriptional regulations

For transcriptional regulations, we obtained TF-target pairs from Transcriptional Regulatory Relationships Unraveled by Sentence-based Text mining (TRRUST) [30] and Human Transcriptional Regulation Interactions database (HTRIdb) [31] and obtained TF-miRNA pairs from mirTrans [32] and TransmiR [33]. As for the data in mirTrans, we reserved the pairs with affinity score no smaller than 1 and conservation score no smaller than 0.95. The TFs to be studied were derived from these four data sources. And then TF-target pairs were divided into TF-gene and TF-TF by the obtained TF, and the TF-TF pairs were removed.

For post-transcriptional regulations, we downloaded the miRNA-target pairs from miRanda [34], PITA [35], and TargetScan [36]. The pairs that appeared at least twice in these three databases were kept. Meanwhile, the miRNA-target pairs were divided into miRNA-TF pairs and miRNA-gene pairs.

Finally, there were 13,768 miRNA-TF pairs, 124,393 miRNA-gene pairs, 53,855 TF-gene pairs and 7036 TF-miRNA pairs, respectively.

#### Disease related genes and miRNAs

We collected disease-related genes from Online Mendelian Inheritance in Man (OMIM) [37] and the Catalogue Of Somatic Mutations In Cancer (COSMIC) [38]. OMIM is a database which collects data, including human genes, genetic phenotypes and the relationships between diseases and genes, while COSMIC is a database which explores the impact of somatic mutations in human cancer. We also collected disease-related miRNAs from miR2Disease [39], PhenomiR [40] and the Human microRNA Disease Database (HMDDv2.0) [41].

At last, we obtained 17 ESCA-related genes and 186 ESCA-related miRNAs, 30 STAD-related genes and 381 STAD-related miRNAs.

#### Gene and miRNA expression profiles

Clinical data and gene/miRNA expression profiles were downloaded from TCGA [42]. First, we retained the samples which satisfied the following three conditions. (1) They should be paired, i.e. there should be a corresponding normal sample for a tumor sample; (2) They should have gene expression profile; (3) They should have miRNA expression profile. We obtained 20 (10 tumor samples and 10 normal samples) and 64 (32 tumor samples and 32 normal samples) samples for ESCA and STAD, respectively.

We filtered the genes whose expression levels were less than 1 in half of the samples. And the miRNAs whose expression levels were missing in greater than 10% of samples were removed. For the remaining miRNAs, we retrieved miRNAs which were related to the specific disease, and then we deleted the miRNAs whose expression levels were missing in more than half of the samples.

After preprocessing, there were 17,150 genes and 471 miRNAs in gene/miRNA expression profiles for ESCA. And there were 17,059 genes and 477 miRNAs in gene/miRNA expression profiles for STAD.

The differential expression analysis is an important way to study the molecular mechanisms, which could help explain the mysteries of organisms. We can obtain differentially expressed molecules using the preprocessed expression data. We used the R package Limma [21] to calculate differentially expressed genes and differentially expressed miRNAs with adjusted *p*-value < 0.05 and |log_2_FC| >1.

For ESCA, we obtained 2769 differentially expressed genes and 105 differentially expressed miRNAs, which contained 1329 down-regulated genes, 1440 up-regulated genes, 17 down-regulated miRNAs, and 88 up-regulated miRNAs, respectively. For STAD, we obtained 3208 and 148 differentially expressed genes and miRNAs, which contained 1752 down-regulated genes, 1456 up-regulated genes, 14 down-regulated miRNAs and 134 up-regulated miRNAs, respectively.

### MiRNA-TF-gene FFL

**Fig. 8:**
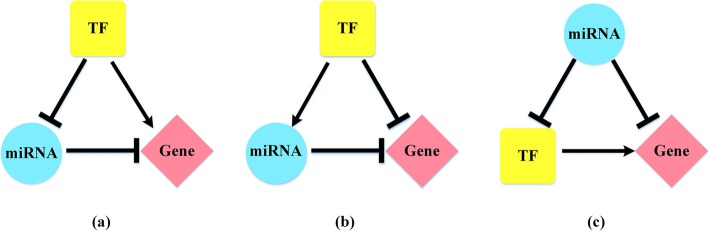
Three categories of MiRNA-TF-gene FFL. **a** TFP-FFL **b** TFN-FFL **c** miRNAN-FFL

The FFL is one of the most important principles in regulating the responses of living cells, in which one TF *A* regulates another TF *B*, while *A* and *B* regulate their common target gene *C* [43]. In this study, we considered two kinds of regulation factors, TFs and miRNAs, so our FFL consists of three elements, one TF, one miRNA and one gene. Besides, we defined the molecule in one FFL regulating the other two molecules as the main regulation factor, and the expression level of the target gene depends on the main regulation factor. As a TF activates or regresses its target, while a miRNA regresses its target, and a TF and a miRNA may regulate mutually, these three elements may constitute multiple categories of FFL models. We focused on three models here (Fig. 8). These three models are really typical on the studies of molecular mechanisms of diseases [44].

We named these three FFLs as TFP-FFL, TFN-FFL and miRNAN-FFL, respectively. As shown in Fig. 8, TFP-FFL describes that a TF inhibits its target miRNA and activates its target gene, and meanwhile the target miRNA inhibits the same target gene. In contrast, TFN-FFL describes that a TF activates its target miRNA and inhibits its target gene, and meanwhile the target miRNA inhibits the same target gene. Similarly, miRNAN-FFL describes that a miRNA inhibits its target gene and inhibits its target TF, meanwhile, the target TF activates the same target gene. The first two models take the TF as the main regulation factor, while the last one takes the miRNA as the main regulation factor. The final effect of TFP-FFL is to up-regulate the expression level of the target gene, while the other two FFLs will down-regulate the expression level of the target gene.

### Random walk with restart

The random walk on a graph describes a walker walks from a current node to one of its neighbors randomly from a certain initial node *s* [45]. When a random walker is allowed to walk from the initial node *s* at each time step with a certain probability *r*, which is called random walk with restart [46].. Random walk with restart (RWR) has been successfully applied in ranking candidate disease genes by walking on biological molecular networks [46–48]. RWR with a restart probability *r* (0 < *r* < 1) is defined as Eq. (1).

1 $$ {p}_{l+1}=\left(1-r\right)\ {Wp}_l+{rp}_0 $$

*W* is a column-normalized adjacency matrix of the network, *p*_*l*_ is a vector in which the *i*-th element holds the probability of being at node *i* at time step *l* [49]. *p*_0_ is an initial vector. Assuming we have *m* seed nodes, in *p*_0_, each seed node has a same initial probability which is 1/*m*, while each non-seed node has zero probability. The whole iteration process will stop when the difference between *p*_*l*_ and *p*_*l* + 1_ is very small, say, less than 10^− 6^.

## Supplementary information

**Additional file 1.** This .xls is a detail description of gene set enrichment analysis for STAD-specific FFL and ESCA-specific FFL

**Additional file 2.** This .xls is a detail description of all candidate molecules

## Data Availability

Gene and miRNA datasets analysed during this study are included in TCGA (https://xenabrowser.net/datapages/) [42]. TF-target pairs were downloaded from TRRUST (https://www.grnpedia.org/trrust/) [30] and HTRIdb (http://www.lbbc.ibb.unesp.br/htri) [31]. TF-miRNA pairs were downloaded from mirTrans (http://mcube.nju.edu.cn/jwang/lab/soft/mirtrans/) [32] and TransmiR (http://cmbi.bjmu.edu.cn/transmir) [33]. MiRNA-target pairs were downloaded from miRanda (http://www.miranda.org/) [34], PITA (https://genie.weizmann.ac.il/pubs/mir07/mir07_dyn_data.html) [35] and TargetScan (http://www.tar-getscan.org/) [36]. Disease-related genes were downloaded from OMIM (http://www.omim.org/) [37] and COSMIC (http://cancer.sanger.ac.uk/cosmic) [38]. Disease-related mi-RNAs were downloaded from miR2Disease (http://www.mir2disease.org/) [39], PhenomiR (http://mips.helmholtz-muenchen.de/phenomir) [40] and HMDDV2.0 (http://210.73.221.6/hmdd) [41].

## Abbreviations

ESCA: Esophageal Carcinoma
FFL: Feed-forward loop
RWR: random walk with restart
SCC: spearman correlation coefficient
STAD: Stomach Adenocarcinoma
TF: transcription factor
